# Direct medical costs of adverse events in Dutch hospitals

**DOI:** 10.1186/1472-6963-9-27

**Published:** 2009-02-09

**Authors:** Lilian HF Hoonhout, Martine C de Bruijne, Cordula Wagner, Marieke Zegers, Roelof Waaijman, Peter Spreeuwenberg, Henk Asscheman, Gerrit van der Wal, Maurits W van Tulder

**Affiliations:** 1 Department of public & occupational health, EMGO Institute, VU University Medical Centre (VUmc), PO Box 7057, 1007 MB Amsterdam, The Netherlands; 2 NIVEL, Netherlands Institute for Health Services Research, PO Box 1568, 3500 BN Utrecht, The Netherlands; 3 Consultant internal medicine, HAJAP, Valeriusstraat 4HS, 1071MH Amsterdam, The Netherlands; 4 Department of Health Economics & Health Technology Assessment, Institute of Health Sciences, Faculty of Earth & Life Sciences, VU University, De Boelelaan 1081, 1081 HV Amsterdam, The Netherlands

## Abstract

**Background:**

Up to now, costs attributable to adverse events (AEs) and preventable AEs in the Netherlands were unknown. We assessed the total direct medical costs associated with AEs and preventable AEs in Dutch hospitals to gain insight in opportunities for cost savings.

**Methods:**

Trained nurses and physicians retrospectively reviewed 7926 patient records in 21 hospitals. Additional patient information of 7889 patients was received from the Dutch registration of hospital information. Direct medical costs attributable to AEs were assessed by measuring excess length of stay and additional medical procedures after an AE occurred. Costs were valued using Dutch standardized cost prices.

**Results:**

The annual direct medical costs in Dutch hospitals were estimated at a total of euro 355 million for all AEs and euro 161 million for preventable AEs in 2004. The total number of hospital admissions in which a preventable AE occurred was 30,000 (2.3% of all admissions) and more than 300,000 (over 3% of all bed days) bed days were attributable to preventable AEs in 2004. Multilevel analysis showed that variance in direct medical costs was not determined by differences between hospitals or hospital departments.

**Conclusion:**

The estimates of the total preventable direct medical costs of AEs indicate that they form a substantial part (1%) of the expenses of the national health care budget and are of importance to hospital management. The cost driver of the direct medical costs is the excess length of stay (including readmissions) in a hospital. Insight in which determinants are associated with high preventable costs will offer useful information for policymakers and hospital management to determine starting points for interventions to reduce the costs of preventable AEs.

## Background

Throughout the world health care workers and policymakers are trying to improve patient safety. The Dutch Patient Safety Research Program[1], showed that adverse events (AEs) affect 5.7% of patients in Dutch hospitals and lead to permanent disability, morbidity and even mortality[2]. Forty percent of these AEs were judged preventable by good clinical practice.

Studies in various countries outside Europe have shown that AEs in hospitals are associated with high direct medical costs [3-9] which have impact on the annual health care budget[5,7,9]. These studies showed that the average excess length of stay (LOS) attributable to AEs ranges from 6 to 8.5 days [3-8] for all AEs. Except for one study[9] which took place over a decade ago, no data on costs of preventable AEs are available.

As health care costs are rising hospitals are forced to reduce costs. Implementation of patient safety improvement interventions in hospitals is often hampered by the investment costs. Thus insight in costs of preventable AEs may help to increase the sense of urgency and prioritise areas to improve patient safety from an economic perspective in addition to the patient and health care perspective.

In this paper we present the total direct medical costs associated with AEs and preventable AEs in Dutch hospitals with regard to subgroups of university and general hospitals. A separate analysis was focussed on AEs occurring in patients who died in hospital. The costs were calculated in two ways, by using the data as determined by the reviewers and with the use of estimation of expected LOS in hospital based on administrative hospital data. In addition, we assessed determinants of AE related costs, such as type of patient admission and AE characteristics, to give insight in the opportunities for cost savings.

## Methods

The instruments of our study were based on the protocol of the Canadian Adverse Event Study, which was originally used by the Harvard Medical Practice Study[3,10]. The design and methods of this study have been described in detail elsewhere[1] a brief summary of the study design and setting is described below.

### Study design and setting

We have performed a retrospective patient record review study in a random, stratified sample of 21 of the 101 Dutch hospitals: 4 university, 17 tertiary teaching and general hospitals. To measure the difference in incidence between hospital types, the sample of hospitals was stratified for hospital type. Proper representation of urban and rural setting in the sample was verified. Eligible hospitals had at least 200 beds, an emergency department and an intensive care unit. A large subsample of deceased hospital patients was included to determine the occurrence of potentially preventable deaths in hospitals more precisely than in previous studies. The power was estimated to detect a difference in AE rates between different hospital types. The parameters in the power calculation were based on the results of the Canadian Adverse Events Study.[3] Assuming an incidence of AEs of 8%, a sample of 4200 hospital admissions of discharged patients and a sample of 4200 admissions of deceased patients were necessary (β = 0.20, α = 0.05) to estimate a 95% confidence interval of 0.5% to both sides.

To measure the difference in incidence between hospital types a selection of 800 hospital admissions per hospital type were necessary to detect a difference from 2% to 3% by an incidence between 3% and 7%. From each hospital, we randomly selected 200 admissions (> 24 hours stay) of discharged patients and 200 (or less if the total of patients who died in 2004 was lower) admissions of deceased hospital patients in 2004, excluding admissions of psychiatry, obstetrics and children < 1 year old. Ethical approval for the study was obtained from the Medical Ethical Research Committee of the VU University Medical Centre.

### Study population

The nursing, medical and, if available, out-patient record of the sampled admissions were reviewed by 66 trained nurses and 55 trained physicians in a three stage review process between August 2005 and October 2006. In the first stage, a nurse screened the records by using 18 screening criteria indicating potential AEs. The 18 screening criteria were:

1. Unplanned admission before index admission (admission reasons are related to the index admission)

2. Unplanned readmission after discharge from index admission

3. Hospital-incurred patient injury (Permanent or temporary injury obtained (acquired) during index admission)

4. Adverse drug reaction

5. Unplanned transfer from general care to (an) intensive care (unit)

6. Unplanned transfer to another acute care hospital (after unexpected deterioration of the patient)

7. Unplanned return to the operating room

8. Unplanned removal, injury or repair of organ during surgery

9. Hospital-acquired infection or sepsis

10. Other patient complication

11. Development of neurological deficit not present on admission

12. Unexpected death

13. Cardiac or respiratory arrest

14. Injury related to abortion or delivery

15. Inappropriate discharge to home

16. Dissatisfaction with care documented in the medical record

17. Documentation or correspondence indicating litigation

18. Any other undesirable outcome not covered above

In the second stage, two physicians independently reviewed the records with one or more positive screening criteria.

For the analysis on patient record level, patients with more than one AE during admission, the AE with the most severe outcome was used for further analysis. Of the initial 8415 sampled records, 8032 were eligible for a first stage review (screening success rate was 95%). Three-hundred eighty three records were unavailable or were inadequate (for example twice sampled or admission was to short). In the second stage 106 records were excluded during screening (for example incomplete documentation or hospitalisation of the patient during the reviewing process)[2]. Finally, 37 (0.5%) hospital admissions could not be linked to the Dutch registration of hospital information (LMR) for linkage to additional information on interventions, due to incorrect unique admission numbers, birth date or sex. This has resulted in 7889 hospital admissions present in the data-analysis.

### Adverse event assessment

The definition of an AE in our study was threefold: an (1) unintended (physical and/or mental) injury which results (2) in temporary or permanent disability, death or prolonged hospital stay and (3) is caused by the health care management rather than the patients' disease[1,11]. A nurse screened the patient records by using 18 screening criteria indicating potential AEs. When one or more screening criteria were found the nurse assigned the patient record to two physicians of the same specialty (general surgeons, general internists, neurologists or paediatricians, depending on the main diagnosis of the admission or age of the patient for the paediatricians) independently reviewed the patient records using an extensive standardised review form. Of each AE the reviewer assessed the impact, location, responsible specialty, clinical procedure, preventability and causes of the AE[1]. If there was disagreement about the presence and/or preventability of an AE between the two independent physician reviews, they started a consensus procedure. If they could not reach consensus, a third trained reviewer gave the final judgement. To determine whether the injury was caused by health care management and the degree of preventability of the AE were measured on a 6-point scale. For AEs a score above three (> management causation more likely, 50/50 but "close call") were considered an AE and for an AE to be preventable a score of 4 and higher (> 50%; preventability more than likely) was needed.

Additional administrative hospital information on diagnosis (ICD-9-DE), secondary diagnoses, acute or non-acute admission, main intervention, secondary interventions, expected LOS, and reason for admission were retrieved for each admission from the LMR maintained by Prismant.

### Cost measurement

The main focus of costs calculated in this study was the health care sector. Only direct medical costs could be taken into account due to the nature and design of this study. Direct medical costs attributable to AEs were assessed by excess length of stay (LOS) during the index admission and re-admission, and by additional medical procedures during the excess LOS as a consequence of the AE.

Excess LOS during the index admission attributable to AEs was calculated by taking the average of the two independent estimates of the physician reviewers and rounded upwards. The excess LOS was determined based on the clinical expertise of the physician reviewers. If they were unable to determine excess LOS, we imputed the excess LOS based on the LMR, notably the difference between expected and observed LOS. The estimates varied from no extra bed days to all bed days of the index admission. For re-admissions attributable to AEs in the index admission we imputed the national average hospital stay in 2004 (7.3 days). Additional medical procedures attributable to AEs were assessed by the physician reviewers, indicating for each AE whether additional interventions or treatment were necessary. Additional information on these interventions was added from the administrative hospital data starting from the date of the AE.

### Cost valuation

The excess LOS and the excess medical procedures were multiplied by unit costs to estimate the total excess direct medical costs. Dutch guideline prices of 2003 were used to estimate the costs of one hospital day of standard or intensive care in a university and general hospital [12-14] (Table 1). Prices were corrected with price-indices for 2004[13,14]. No unit costs for tertiary medical teaching hospitals were available; therefore the guideline prices for general hospitals were used. The unit costs include costs of a standard hospital day, medical and nursing staff, medication, material, equipment, housing and overhead. The costs of procedures and interventions were not included in these costs. The costs of the excess medical procedures were estimated by multiplying the number of procedures by standard price tariffs used for insurance companies, maintained by the Dutch Healthcare Authority.

**Table 1:** Hospital day unit prices 2004

	In euro (corrected for 2004)
Academic hospital day	481,63
General hospital day*	340,99
Intensive care day*	1703,92

* From an economic perspective the costs of tertiary teaching hospitals are the same as general hospitals. Intensive care days are only overall available.

Source: Oostenbrink JB, et al. 2004.

### Statistical analysis

The national weighted incidence of AEs and preventable AEs in Dutch hospitals with 95% confidence intervals (CIs) were calculated, corrected for the overrepresentation of patients admitted to a university hospital and for the overrepresentation of patients who died in hospital, using 'complex samples' option in SPSS 15.0. Detailed information on the weighing of the sampling frame in order to gain representativeness for the Dutch population of hospitalised patients and patients which died during hospitalisation is described in detail previously. [2]

As the unit price of a hospital day in university hospitals is higher than in general hospitals, we analysed the costs attributable to the excess LOS and medical procedures of AEs separately in both groups. Mean total excess costs per hospital admission in which an AE occurred was estimated using weights to adjust for the complex sampling frame. As cost-data were skewed to the right, we used non-parametric bootstrapping, adjusting for the sampling frame, to calculate CIs of cost estimates[15]. Non-parametric bootstrap is the preferred method to estimate 95% CIs around cost-estimates because it uses the distribution of the data rather than assuming a normal distribution. [16]

Moreover, determinants which may influence the costs attributable to AEs, such as age, diagnosis, admission department and medical or surgical responsible specialty, and type of AEs were first analysed univariate. Average costs and 95% CIs which were calculated per subgroup using bootstrapping techniques to resolve the skewed distribution. In addition, a stepwise multivariate multilevel analysis, as data were clustered within hospitals and hospital departments, was used to study the relation of these determinants with the costs of AEs and to test our univariate associations. Within all steps of the multivariate multilevel analysis, data were adjusted by a normalisation procedure for the overrepresentation of university hospitals and deceased patients. The dependent variable in the model was either the costs of AEs or the excess LOS (based on the LMR). Independent variables added to the model were successively age, sex, admission type (urgent or elective), main diagnosis groups (ICD9-DE), Charlson comorbidity index and surgical or non-surgical admission department. The amount of variation in costs of AEs caused by the hospital level or hospital department level was expressed by the intraclass coefficient (ICC).

The national estimate of costs attributable to AEs in 2004 was calculated by multiplying the total amount of Dutch hospital admissions with the AE rate and by subsequently multiplying this with the average costs. The same was done for costs attributable to preventable AEs.

SPSS for Windows version 15.0 was used for most statistical analyses, only the multivariate multilevel analyses were performed using MLwiN version 2.0.

### Sensitivity analysis

As it is sometimes difficult to determine the excess LOS due to the AE based on record review, we used a different approach in calculating excess LOS. For each individual admission in our sample we obtained the expected LOS from the LMR. Within the LMR, for each hospital admission, the expected LOS is calculated based on the following characteristics of the patient and the national mean length of stay that is associated with these characteristics[7]. The characteristics that have been taken into account were:

• Age, divided in 5 classes (0, 1–14, 15–44, 45–64, 65+ years);

• Primary diagnosis. (it included about 1,000 diagnoses classified by the ICD9-CM in three digits);

• Procedures, classified by the Dutch Classification System of Procedures (procedures depend on the diagnosis of the patient. It included five procedure groups)

Together these three parameters produced about 5 * 1,000 * 5 = 25,000 possibilities of expected LOS. Normally these calculations were only used for patients discharged alive, but for the purpose of this study the expected LOS was also calculated for the patients which died in hospital.[17] Excess LOS was calculated as the difference between the expected and the observed LOS.

## Results

### Population

In total 7926 patient records have been reviewed by nurses and physicians. Additional information from the LMR could not be acquired for 37 patients due to record linkage problems. The missing patients were randomly divided over the 21 hospitals. The study population for the following results consisted of 7889 hospital admissions. Characteristics of the 7889 admissions are shown in table 2.

**Table 2:** Comparison of patients with LMR data and all patients reviewed

**Patient data**	Total sample reviewed*	Total sample deceased patients**
Number of inpatient admissions	7889	3959
Number of admissions in university hospitals	1378	780
Mean age in years (sd)	57.5 (21.5)	73.0 (13.4)
Sex (% male)	49.0	53.8
Admission in days mean (SD/median)	8.5 (10.5/5.0)	12.4 (14.3/7.0)
Urgent admissions (%)	53.6	87.0
Admission department in %:		
Surgery	24.1	11.7
Cardiology	12.9	13.0
Internal medicine	15.8	30.2
Orthopaedics	10.4	1.7
Neurology	7.5	12.3
Pulmonary disease	7.2	13.1
Ear, nose and throat	4.3	0.4
Urology	4.2	1.2
Other	13.6	16.4

* Psychiatric and obstetric patients and patients < 1 year were excluded. Day admissions were only excluded in the discharged patients. Figures were weighted for oversampling of deceased patients and of patients admitted to a university hospital.

** Psychiatric and obstetric patients and patients < 1 year were excluded. Figures were weighted for oversampling of deceased patients admitted to a university hospital.

In the 7926 patient records the nurses found one or more screening criteria in 54% of the nursing and medical records. In the second stage the physician reviewers found 774 AEs in 663 hospital admissions. Within these admissions the 663 patients suffered from one or more AE, comprising 216 discharged and 447 patients which died during hospitalisation. The 37 hospital admissions that could not be linked contained no AEs.

### Length of stay

Excess LOS attributable to the AE could be estimated by the physicians in 96% of the cases. In the other cases we imputed the excess LOS based on the data from the LMR. In 22% of the AEs the physician reviewers indicated that the admission itself was caused by the AE, thus the AE occurred in a previous admission but was detected in or was the reason for the index admission. On average, the excess LOS due to the AEs was 10.1 days (95% CI: 6.4 – 11.9) for university hospitals and 8.9 days (95% CI: 6.9 – 9.6) for general hospitals (Table 3). This difference was not statistically significant.

**Table 3:** Excess length of stay and mean costs of AEs for (potentially preventable) adverse events in the Netherlands in 2004

	**Mean excess LOS in days (95% CI)**	**Mean costs of LOS in € (95% CI)**	**Mean costs of medical procedures in € (95% CI)**	**Mean total costs of AEs in € (95% CI)**
All AEs* (n = 456)	**9.1 (7.6 – 10.5)**	**3852 (3081 – 5009)**	**703 (452 – 923)**	**4555 (3694 – 5790)**

Deceased patients**	6.3 (5.3 – 8.3)	3962 (2582 – 4179)	524 (366 – 704)	4487(3065 – 4784)
University hospitals***	10.1 (6.4 – 11.9)	5298 (3580 – 7943)	189 (53 – 282)	5487 (3673 – 8075)
General hospitals***	8.9 (6.9 – 9.6)	3543 (2695 – 4273)	813 (556 – 969)	4356 (3357 – 5062)

All preventable AEs* (n = 182)	**10.3 (7.8 – 13.2)**	**4778 (3081 – 7066)**	**507 (309 – 850)**	**5286 (3470 – 7786)**

Deceased patients**	6.5 (4.4 – 9.7)	3763 (2266 – 4778)	765 (338 – 952)	4528 (2830 – 5370)
University hospitals***	15.7 (8 – 21)	8054 (5041 – 11834)	45 (0 – 173)	8100 (5144 – 11944)
General hospitals***	9.7 (7.4 – 13.2)	4436 (2609 – 7396)	556 (323 – 931)	4992 (3033 – 8188)

* Psychiatric and obstetric patients and patients < 1 year were excluded. Day admissions were only excluded in the discharged patients. Figures were weighted for over sampling of deceased patients and of patients admitted to a university hospital.

** Subgroup analysis, sample is not weighted for overrepresentation of deceased patients in the sample.

*** Psychiatric and obstetric patients and patients < 1 year were excluded. Day admissions were only excluded in the discharged patients. Figures were weighted for over sampling of deceased patients.

### Medical procedures

The medical procedures attributable to AEs, mainly re-operations, accounted for approximately 15% of the medical costs attributable to AEs (Table 3). Non-invasive procedures (for example casting a leg, pulmonary function tests or X-thorax) were almost never recorded in the registration of hospital information. The registration of medical procedures other than surgical procedures in the registration of hospital information was poor in both university and general hospitals. Consequently, the estimated costs of medical procedures are an underestimation of the real costs.

### Costs of (preventable) AEs

Although the number of preventable AEs in university hospitals was lower than in general hospitals[2], the total costs rose substantially when the AE was reviewed as preventable (Table 3). This result however was not statistically significant.

### Determinants of costs of (preventable) AEs

The excess LOS (5.6 days to 16.8 days) and consequently the excess costs (€2979 to €6649) of preventable AEs increased when the impairment or disability was more severe (Table 4). In hospital deaths, in which readmissions were impossible, the average costs of AEs were somewhat lower than in discharged patients. Surgical AEs resulted in longer excess LOS and thus higher excess costs than non-surgical AEs. Internal medicine en neurology costs increased when the AE was preventable, costs and excess LOS of the preventable AEs in orthopaedics decreased. With respect to the types of AEs, the surgical and medication related AEs were the most expensive. The relative importance of subgroups changed when adding the costs to the distribution. For example the impact of the age group 65–79 changed from 32% by just looking at AEs to 57% by combining preventable AEs and costs of preventable AEs and the preventable AEs with an intensive care admission only contributed to 3% of all preventable AEs, while the costs as a result of these preventable AEs resulted in almost 30% of all preventable AE costs.

**Table 4:** Univariate estimation of excess length of stay and excess costs (in Euro) of adverse events

Cost categories	All Adverse events* (n = 663, weighted n = 456)			Preventable Adverse events* (n = 283, weighted n = 182)		
Disability**	N	Mean days (se)	Cost in € including interventions (se)	N	Mean volume (se)	Cost in € (se)

No disability	115	5.5 (1.0)	2970 (475)	29	5.6 (1.7)	2979 (781)
Minor disability	276	10.4 (0.7)	5174 (598)	117	11.2 (1.2)	5973 (1332)
Permanent disability	21	16.0 (3.1)	6232 (1331)	16	16.8 (3.9)	6649 (1667)
Deceased	35	7.8 (2.8)	4566 (1636)	17	7.0 (5.0)	3831 (2263)

**Speciality**						

**Surgical**	293	10.1 (0.7)	5474 (601)	115	10.7 (1.3)	6122 (1371)
Medical	163	7.4 (0.8)	2909 (339)	66	9.7 (1.6)	3831 (654)

**Type of hospital**						

Academic	80	10.1 (1.5)	5488 (858)	17	15.7 (3.3)	8100 (1515)
General hospitals	376	8.9 (0.6)	4356 (460)	165	9.7 (1.1)	4992 (985)

**Age categories**						

1–18 years	10	1.4 (0.4)	597 (127)	2	0 (0)	0 (0)
19–40 years	54	12.2 (1.9)	5303 (854)	22	12.3 (3.3)	4727 (1280)
41–65 years	161	7.1 (0.8)	3736 (379)	61	8.2 (1.5)	3652 (537)
66–79 years	139	11.9 (1.2)	6413 (1170)	55	14.8 (2.2)	9290 (2770)
80 years and over	91	7.5 (1.0)	3134 (407)	42	6.9 (1.5)	2923 (700)

ICD9DE primary groups***						

Neoplasms	52	8.8 (2.1)	7636 (2923)	13	14.4 (6.8)	18734 (10772)
Circulatory system	86	7.5 (1.0)	4396 (608)	26	10.7 (2.2)	4542 (853)
Respiratory system	31	6.1 (1.7)	2519 (751)	18	7.0 (2.8)	2938 (1235)
Digestive system	45	10.4 (1.9)	4789 (951)	20	9.3 (3.3)	3963 (1179)
Genitourinary system	35	8.5 (1.3)	4068 (916)	13	13.7 (2.7)	6640 (2250)
Muscoskeletal system and connective tissue	41	7.4 (1.6)	3093 (553)	16	3.1 (0.7)	1601 (330)
Symptoms, signs, and ill-defined conditions	33	7.8 (1.4)	2834 (484)	13	11.1 (2.8)	3831 (981)
Injury and poisoning	69	14.4 (1.8)	6084 (781)	31	14.2 (2.9)	5976 (1172)

Admission departments***						

Cardiology	54	8.8 (1.7)	3852 (766)	18	9.7 (2.2)	3685 (787)
Surgery	146	10.7 (1.0)	5443 (455)	59	12.2 (2.0)	4962 (746)
Intensive Care	10	16.0 (7.8)	29307 (14313)	5	28.3 (13.7)	52058 (25329)
Internal Medicine	68	8.8 (1.5)	3423 (595)	15	13.4 (4.7)	5451 (1906)
Pulmonary diseases	31	7.9 (2.6)	3137(955)	22	7.7 (3.5)	3010 (1251)
Neurology	21	7.3 (2.3)	2700 (1002)	13	10.8 (3.5)	4057 (1572)
Orthopaedic	42	9.0 (1.5)	3739 (532)	22	4.4 (0.8)	2362 (317)
Urology	26	9.5 (1.0)	3854 (375)	8	11.3 (1.6)	4336 (703)

**Type of AE**						

Diagnostics	29	12.0 (2.8)	4812 (1250)	24	13.4 (3.2)	5433 (1444)
Surgical	249	10.3 (0.8)	5764 (695)	84	10.8 (1.7)	6735 (1854)
Intervention (non surgical)	81	6.0 (0.8)	2414 (337)	26	9.6 (1.9)	3454 (706)
Medication	66	8.1 (1.4)	3105 (579)	21	9.2 (2.9)	3992 (1309)
Other clinical activities	16	5.1 (1.6)	2438 (757)	13	6.0 (1.8)	2832 (856)
Discharge	6	18.5 (6.9)	6856 (2477)	6	18.5 (6.9)	6856 (2478)
Other	11	5.2 (2.0)	2828 (1545)	9	2.9 (1.6)	2131 (1740)

* Figures were weighted for oversampling of deceased patients and of patients admitted to a university hospital.

** Unable to determine in 9 and 3 patients in respectively all AEs and preventable AEs

*** Largest ICD-9-DE groups only

The multivariate multilevel analysis showed that the height of the costs attributable to AEs was statistically significant higher for patients with a higher age, surgical patients compared to non-surgical patients, and several diagnostic groups (table 5). After adding the diagnostic groups to the model and after correction for the previous added variables AEs in patients with neoplasms were more expensive followed by circulatory diseases, respiratory diseases, diseases of the digestive system, symptoms, signs and ill-defined diseases and injury and poisoning. Variation at hospital and hospital department level showed little influence on variance of costs, respectively ICC = 0.006 and ICC = 0.008.

**Table 5:** Results of the multivariate multilevel analysis of the costs of AEs with clustering on hospital level and hospital department level

	All adverse events β	Preventable adverse events β
Intercept	366	289
Adverse event	4516	3521
Age	-11.2 *	-9.6 *
Sex	-3.3	-7.5
Admission type	-341	-506
**Diagnostic groups (ICD9DE)**		
Endocrine, nutritional and metabolic diseases, and immunity disorders	-584	-568
Nervous system and sense organs	-474	-531
Circulatory system	-1735 *	-1727 *
Respiratory system	-1334 *	-1346 *
Digestive system	-1049 *	-986 *
Genitourinary system	-606	-608
Symptoms, signs and ill-defined conditions	-872 *	-909 *
Injury and poisoning	-1283 *	-1162 *
All other codes	-896 *	-898 *
Charlson index	-81	-81
Speciality	-676 *	-606 *

Dependent variable: costs of admission. Independent variables: age: continuous variable, sex: male as reference, admission type: non urgent as reference, diagnostic groups: neoplasms as reference, Charlson comorbidity index: continuous variable, speciality: non-surgical as reference

### Extrapolation

The excess LOS attributable to preventable AEs (10.3 days) was higher than the mean duration of a hospital stay in 2004 (7.3 days). Extrapolating these figures results in an estimate of 31,164 hospital admissions in which a preventable AE occurred or the entire admissions was caused by an AE and in 320,680 bed days attributable to preventable AEs in the Netherlands in 2004.

Medical costs attributable to AEs during hospitals admissions in 2004 were more than € 355 million (95% CI: €316 million – €398 million) for all AEs and more than €161 million (95% CI: €134 million – €195 million) for preventable AEs. The national health care budget in 2004 was €14.5 billion therefore all AEs resulted in 2.4% and preventable AEs 1.1% of the national health care budget.

### Sensitivity analysis of costs of AEs

In the sensitivity analysis the excess LOS is calculated by subtracting the observed LOS with the expected LOS based on national reference values. The analysis of observed LOS and expected LOS showed a lower, but trend wise similar, result as the analysis based on reviewers excess LOS. The mean excess LOS was 5.9 days and the total costs of AEs (including readmissions and procedures) €4,446 per AE. The mean excess LOS of preventable AEs was 4.4 days and the total costs €3,634 per preventable AE. Within admissions without AEs the observed LOS was slightly lower, patients are on average 0.5 days shorter in hospital than expected.

## Discussion

Extrapolation of the results of this study showed that the total potentially preventable direct medical costs of AEs in Dutch hospitals are more than €161 million in Dutch hospitals in 2004; this is approximately 1% of the annual hospital budget of €14.5 billion. [18] The cost driver of the direct medical costs is the excess LOS in the hospital. Unit price of ICU stay, surgical AEs and age influence the impact of the direct medical costs in addition to incidence of AEs alone. We have also seen that, although it is often assumed that patients who die, whether or not related to an AE, have no or low excess costs, our study showed that the excess costs related to (preventable) AEs in patients who die in hospital are still substantial both for excess LOS and extra interventions. When disregarding the readmissions of the discharged patients, the excess LOS related to the AE was almost as high for deceased compared to discharged patients (6.3 days and 7.4 days respectively). These results are in concordance with the results of the cost of illness studies preformed in the Netherlands. Health care costs are ten-times more expensive in the year before death but decline when people die at older age[19].

There are several reasons to assume that our cost estimation is conservative. Firstly, other research has shown that due to inefficiencies after things go wrong, the average costs of a hospital day becomes more expensive after an AE[20]. We used the same guideline unit price of a hospitalisation day before and after an AE. Secondly, non-invasive procedures are underreported in the LMR, where surgical interventions are registered well. Since non-invasive procedures are no cost-drivers, we do not expect this has led to major distortion of our results. Thirdly, as some AEs do not become apparent until after the selected admission, we also took into account the hospitalisation after the selected admission when related to the AE. When the physicians reported a hospitalisation after the index admission they did not record the number of readmissions and the number of days of each readmission. Therefore only one readmission was added to the excess LOS with an average LOS according to the Dutch reference values (7.3 days in 2004). Fourth, due to the retrospective study design and use of administrative information, the information of each admission may not have been complete. As a result the reviewers of the patient records may have missed some relevant data. Consequently, our estimate might be an underestimation of the total direct medical costs of AEs. However, knowing the outcome and its severity may influence judgment of the AE and the excess LOS. Thus, the possibility to reduce costs as a result of preventable AEs may have been overestimated by retrospective analysis of the AEs[21].

Although it is sometimes difficult to attribute the excess LOS or the additional procedures to the AE, the two approaches we used to calculate the total expenses showed similar results. Where the excess LOS based on the reviewers viewpoint may be an overestimation of the true excess LOS, the difference between the expected and observed LOS based on the LMR data may be an underestimation. The expected LOS is an estimation based on yearly data including admissions with AEs. The use of observed LOS minus the expected LOS may lead to an underestimation of the excess LOS. Nevertheless, despite these limitations, excess LOS gives a useful estimate of the direct medical costs associated with AEs and the potential cost savings of preventable AEs. Moreover because the admissions without an AE show on average no excess LOS (half a day shorter than expected). Patients with an AE stayed in hospital on average 4.6 more days than expected. The LMR is not an economic database, but an administrative database, so there are reasons to assume registration bias. Nevertheless, results of internal validation of our data showed that the registered information in the LMR provided by the hospitals was in good accordance with the information from the patient records. With respect to the most important data such as LOS, admission department and main diagnosis the results were good.

We used price indices appropriate for cost estimates determined from a societal perspective although the perspective of our study was focussed on the hospital. Since there were no cost estimates available from a hospital perspective and the most important elements are included in the standard unit price of a hospital day[13], we do not assume that a different perspective would have led to different results.

A recent study has shown that yearly €85 million of health care costs were caused by potentially preventable hospital admissions related to medication related adverse events in outpatient care[22]. As our study only focuses on the AEs during or caused by hospitalisation, the results of that study can be added to our results when looking at patient safety issues in the Netherlands.

Our study only focuses on the direct medical costs of AEs in hospital. From a societal perspective outpatient health care, loss of income and premature death are also important and the estimation of costs related to AEs would have been higher when estimated from a societal perspective.

Although judgement of presence of AEs is difficult, retrospective patient record studies currently offer the best method available to assess incidence of AEs[23]. By making use of the three stage AE determination method, the representative sample of both hospitals and number of patient records our study produced the needed insight in the present state of patient safety in hospitals[1]. Moreover, the additional information from each hospital admission from the LMR gave us an opportunity to ground our results.

## Conclusion

The estimates of the total potentially preventable direct medical costs of AEs indicate that they form approximately 1% of the expenses of the national health care budget. The cost driver of the direct medical costs is the excess LOS in the hospital.

Despite the limitations by retrospective analysis of AEs, the costs of AEs in the Netherlands, estimated in this study are high and show that improvement of patient safety by preventing AEs may be cost saving. If the financial system is such that the hospital or hospital department profits of this cost reduction, it may offer an economic incentive to invest in patient safety. Insight in the determinants of potentially preventable AEs offers a starting points for patient safety interventions.

## Abbreviations

AE: adverse event; ICC: Intraclass coefficient; ICD-9-DE: Dutch version of the International Classification of Diseases, Ninth revision, Clinical Modification (ICD-9-CM); LMR: Dutch registration of hospital information; LOS: length of stay

## Competing interests

The authors declare that they have no competing interests.

## Authors' contributions

LH wrote the manuscript, prepared the study protocol and instruments, and collected and analyzed data. MB wrote the manuscript, is the general supervisor of the retrospective record study, contributed to the design and conception of the study, and coordinated the study. CW is the general supervisor of the Dutch patient safety research program, contributed to the design and conception of the study, and contributed to the manuscript. MZ collected data and contributed to the manuscript. RW prepared the review forms within a web-based program, managed the acquisition of data, and contributed to the data analysis and to the manuscript. PS analyzed the data and contributed to the manuscript HA contributed to the manuscript and reviewed patient records. GW contributed to the design and conception of the study and has been involved in revising the article critically for important intellectual content (former general supervisor of the Dutch patient safety research program and research group). MT contributed to the design of the study and contributed to the manuscript. All authors gave their approval of the final version of the manuscript.

## Pre-publication history

The pre-publication history for this paper can be accessed here:


